# Idiopathic True Aneurysms of the Brachial Artery: A Short Case Series and Scoping Review

**DOI:** 10.3390/jcm15010295

**Published:** 2025-12-30

**Authors:** Maria Leonida, Spyros Papadoulas, Melina S. Stathopoulou, Andreas Tsimpoukis, Chrysanthi Papageorgopoulou, Konstantinos Nikolakopoulos, Nikolaos Krinos, Aliki Skandali, George Theofanis, Andreas Antzoulas, Dimitrios Litsas, Panagiotis Dimitrios Papadopoulos, Petros Zampakis, Ioannis Maroulis, Vasileios Leivaditis, Francesk Mulita

**Affiliations:** 1 Department of Vascular Surgery, General University Hospital of Patras, University of Patras Medical School, Rio, 26504 Patras, Greece; m.leonidaa02@gmail.com (M.L.); melinastath@gmail.com (M.S.S.); tsimpoukis.and@gmail.com (A.T.); chrisanthi.papageorg@gmail.com (C.P.); konstantinosn@yahoo.com (K.N.); nickkrinos@yahoo.gr (N.K.); alsk280502@gmail.com (A.S.); 2 Department of Surgery, General University Hospital of Patras, University of Patras Medical School, Rio, 26504 Patras, Greece; gtheofanis3@yahoo.gr (G.T.); a.antzoulas@hotmail.com (A.A.); maroulisupatras@gmail.com (I.M.); 3 Department of General Surgery, General Hospital of Lamia, 35100 Lamia, Greece; dimlitsas@icloud.com; 4 Department of Surgery, Spital Herisau, 9100 Herisau, Appenzell Ausserrhoden, Switzerland; panospapado1997@icloud.com; 5 Department of Radiology, General University Hospital of Patras, University of Patras Medical School, Rio, 26504 Patras, Greece; pzampakis@gmail.com; 6 Department of Cardiothoracic and Vascular Surgery, Westpfalz Klinikum, 67655 Kaiserslautern, Germany; vnleivaditis@gmail.com

**Keywords:** brachial artery aneurysm, true aneurysm, peripheral aneurysm, atherosclerosis

## Abstract

**Background:** Brachial artery aneurysms are a rare entity occurring sporadically at all ages. Common causes are trauma, infection, connective tissue disorders, genetic syndromes, immunosuppression, and a history of arteriovenous vascular access. Pseudoaneurysms are mainly of traumatic or iatrogenic origin. Idiopathic true brachial artery aneurysms are even scarcer, attributed to inherited susceptibility for aneurysm formation or to atherosclerosis. Due to the rarity of these aneurysms, we report our experience along with a current literature review. **Methods:** A retrospective search was conducted in the Vascular Surgery Department database of a tertiary referral center for vascular surgery, covering procedures from January 1991 to October 2025. Patients were included if they had undergone idiopathic true brachial artery aneurysm repair. Clinical records, operative details, imaging studies, and follow-up data were reviewed. We additionally provide a literature review regarding clinical presentation, signs, pathophysiology, diagnosis, and treatment of these aneurysms. **Results:** Amongst all procedures performed for aneurysmal repair, in the searched period, we identified three patients who met these criteria. All three underwent successful elective operations. Individual characteristics of the retrieved cases are reported. **Conclusions:** The open repair of true idiopathic brachial artery aneurysms is a technically simple approach that leads to satisfactory outcomes. Endovascular therapy is technically unfavorable in this type of aneurysm.

## 1. Introduction

Brachial artery aneurysms (BAAs) are a rare entity that accounts for approximately 0.5% of all peripheral artery aneurysms [1,2]. They may present in the form of a true aneurysm or a pseudoaneurysm [3]. Usual causative factors such as trauma, infection, connective tissue disorders, genetic syndromes, immunosuppression, and history of arteriovenous vascular access (AVF) are absent in idiopathic true BAAs [4,5,6,7,8]. The causative process in these cases may be attributed to atherosclerosis and to inherited (familial) susceptibility for aneurysmal degeneration [9,10]. Degenerative atheromatous aneurysms are often associated with cardiovascular risk factors [11,12]. Most are fusiform, but saccular morphology does not exclude the diagnosis of a true aneurysm, as all three layers may be present [13,14,15,16]. These aneurysms may remain asymptomatic or present with complications like thrombosis, distal embolism, and rarely rupture [4,17]. Treatment is essential not only for symptomatic but also for asymptomatic individuals to prevent complications [4].

We present a series of three patients with idiopathic true BAAs treated in our institution. They underwent aneurysm excision and reconstruction with reversed basilic vein interposition. We additionally performed an extensive literature review regarding idiopathic true BAAs. Aneurysms in the context of congenital syndromes, trauma, infection, connective tissue disorders, arteriovenous malformations, immunosuppression, and a history of AVF were excluded.

Given the rarity of idiopathic true brachial artery aneurysms and the heterogeneity of available reports, we conducted a scoping review to map the existing literature, describe clinical presentations, diagnostic approaches, and management strategies, and identify knowledge gaps. In accordance with PRISMA-ScR methodology, no formal risk-of-bias assessment, quantitative synthesis, or meta-analysis was planned or performed.

## 2. Materials and Methods

### 2.1. Study Design

This study consists of two components:

- a retrospective single-center case series of patients treated for idiopathic true brachial artery aneurysms, and
- a scoping review of the literature, conducted in accordance with the PRISMA Extension for Scoping Reviews (PRISMA-ScR) guidelines.

### 2.2. Institutional Case Series

A retrospective search of the electronic medical records and operative database of the Department of Vascular Surgery, General University Hospital of Patras in Greece, was performed. The software was Rama 2.3 (2024). The study period extended from January 1991 to October 2025. Patients were included if they underwent open surgical repair of a true brachial artery aneurysm during the study period. No additional exclusion criteria were applied within this category.

A structured search of the institutional operative database was performed using the keywords ‘brachial artery aneurysm’, ‘upper extremity aneurysm’, and ‘peripheral arterial aneurysm’. This search identified all patients who underwent surgery for a brachial artery aneurysm during the study period.

**Figure 1:**
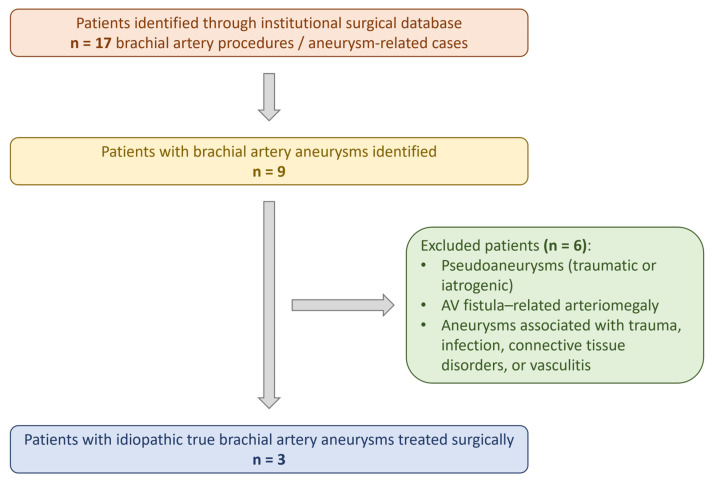
Institutional patient selection flowchart. Flow diagram illustrating identification and selection of patients from the institutional surgical database, including excluded cases and final inclusion of three patients with idiopathic true brachial artery aneurysms treated surgically.

The designation of aneurysms as idiopathic was based on the absence of recognized etiological factors, including trauma, infection, arteriovenous fistulas, vasculitis, connective tissue disorders, or iatrogenic injury, as documented in the clinical records and operative findings. Common cardiovascular comorbidities, such as hypertension, were not considered exclusion criteria, as they are not established primary causes of true brachial artery aneurysm formation. Patients with pseudoaneurysms, aneurysms related to trauma, infection, connective tissue disorders, vasculitis, congenital syndromes, arteriovenous fistulas, immunosuppression, or iatrogenic causes were subsequently excluded. After application of these criteria, three patients with idiopathic true brachial artery aneurysms treated surgically at our institution were identified and included in the case series. Demographic data, clinical presentation, imaging findings, operative details, and early postoperative course were reviewed. The institutional patient identification and selection process is summarized in Figure 1 which illustrates the total number of brachial artery–related cases identified, the reasons for exclusion, and the final inclusion of three patients with idiopathic true brachial artery aneurysms treated surgically.

### 2.3. Literature Search Strategy

A structured literature search was performed in the following electronic databases: PubMed (MEDLINE), Scopus, Web of Science, and the Cochrane Database of Systematic Reviews. The search covered all articles published in English up to October 2025.

The following keywords and Boolean combinations were used:

- “brachial artery aneurysm”;
- “true brachial artery aneurysm”;
- “idiopathic brachial artery aneurysm”;
- “upper extremity aneurysm”;
- “peripheral arterial aneurysm”.

Reference lists of relevant articles were manually screened to identify additional eligible studies.

### 2.4. Study Selection and Eligibility Criteria

Two authors independently screened titles and abstracts for relevance. Full-text articles were subsequently assessed for eligibility. Disagreements were resolved by consensus.

Studies were included if they:

- Reported true aneurysms of the brachial artery;
- Were classified as idiopathic, with no identifiable secondary cause;
- Provided clinical, imaging, and/or operative data.

Studies were excluded if they involved:

- Pseudoaneurysms;
- Aneurysms related to trauma, infection, connective tissue disorders, vasculitis, congenital syndromes, or arteriovenous fistulas;
- Non-brachial upper extremity aneurysms;
- Non-English publications or studies without accessible full text.

### 2.5. Data Extraction and Synthesis

Data extracted included patient demographics, clinical presentation, aneurysm characteristics, diagnostic imaging, treatment modality, conduit type, and reported outcomes. Due to heterogeneity and the rarity of the condition, results were synthesized descriptively. In keeping with the exploratory and descriptive nature of a scoping review, no risk-of-bias assessment, quantitative synthesis, or meta-analysis was performed.

The scoping review was conducted in accordance with the PRISMA Extension for Scoping Reviews (PRISMA-ScR), and the completed checklist is provided as Supplementary Material.

### 2.6. Statistical Analysis

Descriptive statistics were used to summarize data from the scoping review. Continuous variables are reported as means and ranges, while categorical variables are presented as absolute numbers and percentages.

All analyses were performed only on cases for which the relevant data were reported. No data imputation was performed. The denominator for each variable, therefore, varies and is explicitly stated where applicable. Cases in which specific variables were not reported were categorized as “not reported (NR)” and excluded from calculations for that variable.

Given the rarity of idiopathic true brachial artery aneurysms and the heterogeneity of the available reports, no inferential statistical analysis or meta-analysis was attempted, in accordance with the descriptive nature of a scoping review.

## 3. Case Series

This study was conducted in conformity with ethical standards and guidelines. Ethical approval for the research was obtained from the institutional review board of the University Hospital of Patras (IRB no. 37/3 November 2025). The study adheres to the principles outlined in the Declaration of Helsinki regarding research involving human subjects. Written informed consent was obtained from all three patients for the use of their clinical data.

We performed a comprehensive and structured search of the medical database of the Department of Vascular Surgery, General University Hospital of Patras, Patras Medical School. It includes identification codes, operative reports, radiological images, and information for all surgical procedures. The search period extended from January 1991 to October 2025. The primary search keywords employed were “brachial artery aneurysm”, “true brachial artery aneurysm”, “peripheral aneurysm”, “upper limb aneurysm”, and “upper extremity aneurysm”. Patients were included if they met the following criteria: (i) referring for vascular reconstruction; (ii) undergoing open repair of a brachial artery aneurysm; (iii) complete clinical and radiological data.

Amongst all procedures performed for aneurysmal repair, in the searched period, we identified three patients who met these criteria. All three underwent successful elective operations. Individual characteristics of the retrieved cases are reported below and summarized in Table 1.

### 3.1. Case 1

A 28-year-old male patient presented with an asymptomatic right BAA, 3.5 cm in diameter, diagnosed with color duplex ultrasound (CDU). It was in the distal third of the brachial artery (BA), extending slightly to the ulnar artery. No aneurysms at other sites were present. Under general anesthesia, the aneurysm was excised, and reconstruction with a reversed basilic vein interposition was performed between the brachial and the radial artery. The ulnar artery was ligated. Histopathological examination revealed a true brachial artery aneurysm. The postoperative course was uneventful.

### 3.2. Case 2

A 46-year-old male patient presented with an asymptomatic left brachial artery aneurysm, measuring 4 cm in diameter. Color duplex ultrasonography demonstrated a fusiform aneurysm located in the distal third of the brachial artery, extending to the brachial artery bifurcation. No aneurysms at other arterial sites were identified. Under general anesthesia, the aneurysm was excised, followed by arterial reconstruction using a reversed great saphenous vein interposition graft. The distal anastomosis was performed at the level of the brachial artery bifurcation, and the length of the venous graft was approximately 10 cm. Histopathological examination confirmed a true brachial artery aneurysm. The postoperative course was uneventful, with preserved distal pulses.

### 3.3. Case 3

**Figure 2:**
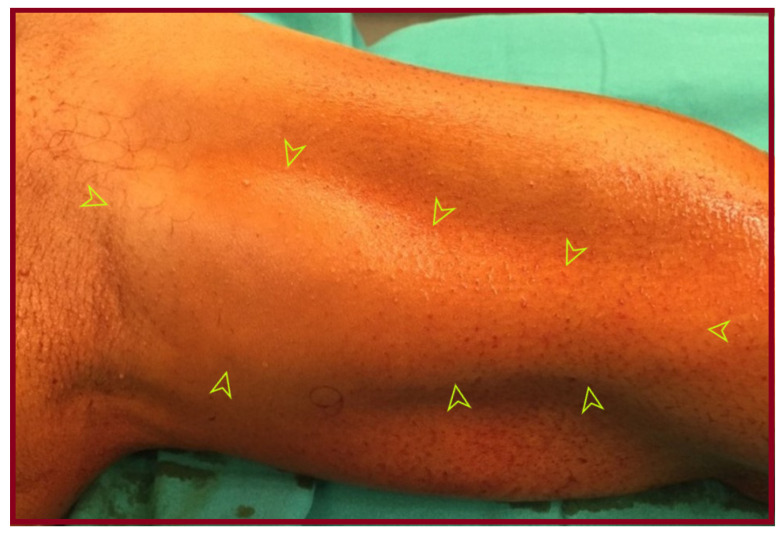
The patient presented with a visible dilatation at his left upper arm, due to a pulsatile mass (arrows).

**Figure 3:**
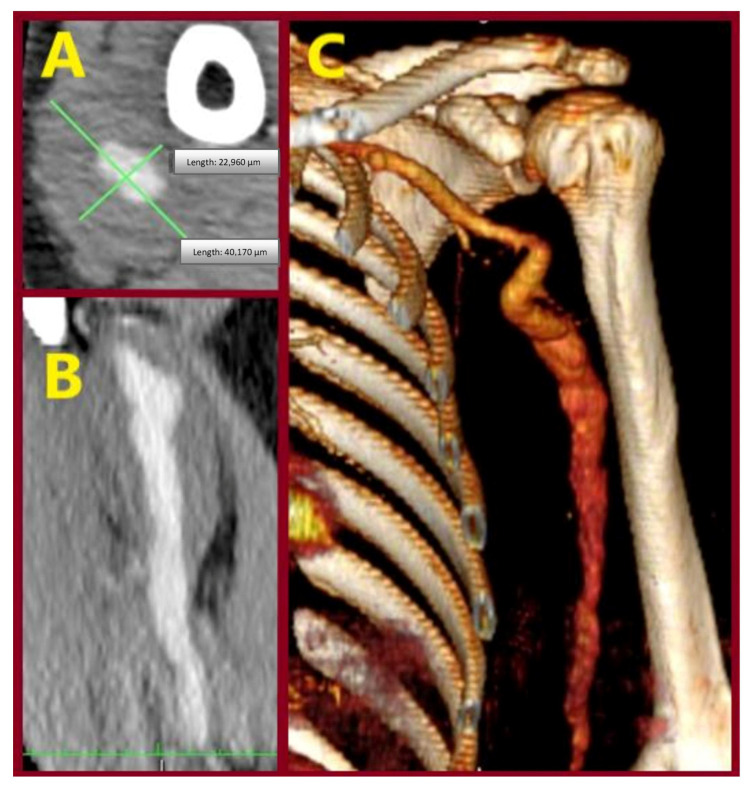
Computed tomography angiography depicting the brachial aneurysm. (**A**) Axial image, (**B**) coronal image, (**C**) 3-D reconstruction.

**Figure 4:**
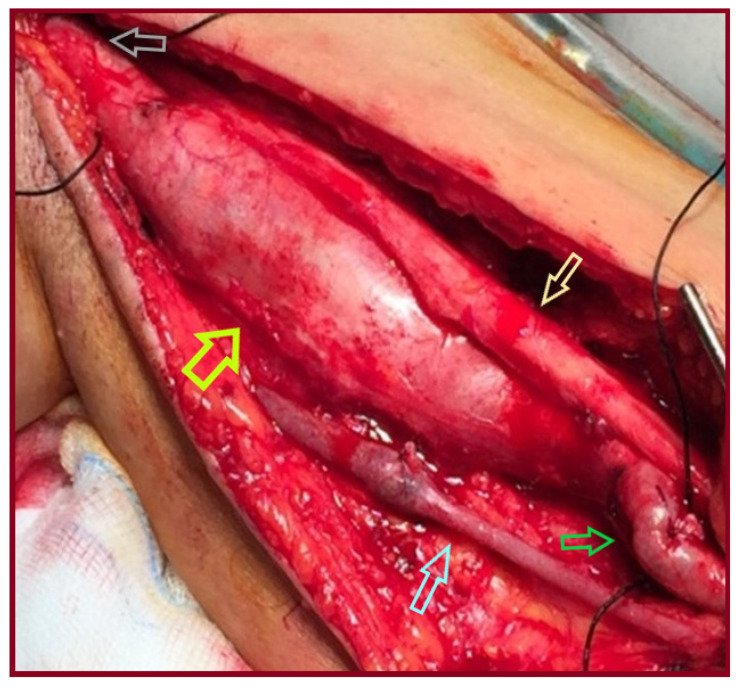
The brachial aneurysm after surgical dissection (gray arrow: axillary artery, light green arrow: the aneurysm’s sac, green arrow: the distal brachial artery, light blue arrow: the basilic vein, yellow arrow: the median nerve).

**Figure 5:**
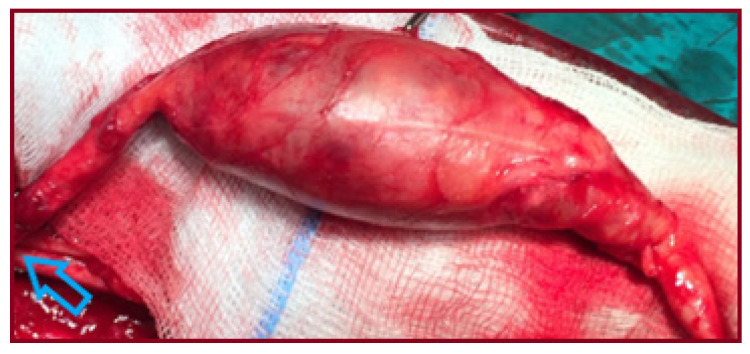
The brachial aneurysm after complete dissection (blue arrow: axillary artery).

**Figure 6:**
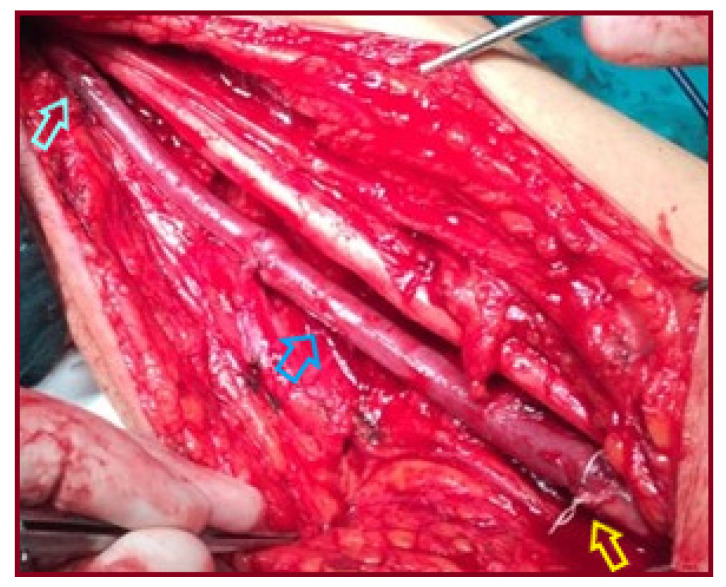
A reversed basilic vein graft was interposed after aneurysm resection (light blue arrow: the proximal anastomosis, blue arrow: the venous graft, yellow arrow: the distal anastomosis).

**Figure 7:**
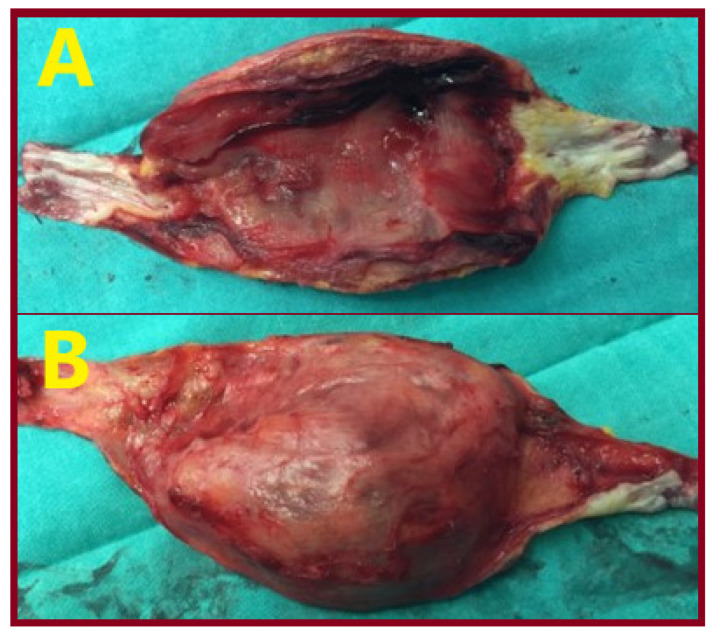
The specimen with the aneurysm’s sac opened. (**A**) Frontal view, (**B**) posterior view.

A 65-year-old male patient presented with an asymptomatic swelling in the left upper arm (Figure 2). Physical examination revealed a pulsatile mass with normal distal pulses. CTA depicted a BAA, 4 cm in diameter (Figure 3). The patient denied any history of trauma, infection, arteriovenous fistula creation, or aneurysmal disease in his first-degree relatives. CDU Imaging excluded an aneurysm at other sites. Under general anesthesia, surgical excision of the aneurysm was performed, followed by a vascular reconstruction using a reversed basilic vein graft. End-to-end anastomoses were completed proximally and distally (Figure 4, Figure 5 and Figure 6). Immediate postoperative assessment confirmed intact radial and ulnar pulses. Histopathological examination of the specimen revealed a true brachial artery aneurysm with atherosclerotic changes (Figure 7). The patient’s postoperative course was uneventful.

Each case represents a distinct patient with separate operative records, imaging findings, and histopathological confirmation.

## 4. Discussion and Literature Review

Diagnosis of an upper extremity arterial aneurysm was first set by Hippocrates in 460 BC [18]. Aneurysm is defined by a 50% focal arterial dilatation, while a true aneurysm maintains all three wall layers [11,19]. On the contrary, the sac of pseudoaneurysms consists mainly of fibrous tissue [20]. True aneurysms are mostly fusiform, and pseudoaneurysms are mainly saccular. Some true aneurysms may be present in the context of diffuse aneurysmal disease [20]. They may be congenital or acquired. In children, they may be associated with underlying conditions such as Kawasaki disease, giant cell arteritis, polymyarteritis, Horton or Takayasu arteritis, fibromuscular dysplasia, Bechet’s disease, cystic adventitial disease, or connective tissue disorders (Ehlers-Danlos, Marfan or Loys-Dietz syndromes, Neurofibromatosis type 1), Menkes disease, and Kaposi’s sarcoma [11,20,21,22,23,24,25,26,27,28]. There are nine types of childhood aneurysms, classified by etiology and pathogenesis, based on the Ann Arbor classification [25]. Interestingly, congenital-idiopathic aneurysms in the absence of any systemic disease are categorized as Class VIII. They may involve the abdominal aorta, axillary, brachial, or iliofemoral arteries [25]. A rheumatologist and geneticist should guide the laboratory work-up for associated conditions in children [29]. Buerger’s disease and true BAA have also been described [30]. The natural history of BAAs is not well defined [25]. Due to the rarity of idiopathic true BAAs, no universal treatment guidelines exist [4]. More research is required to better illuminate the causes, progression, and best treatment of BAAs [1].

We performed a descriptive synthesis of the literature and searched medical databases, including PubMed, Scopus, Web of Science, and the Cochrane Database of Systematic Reviews. The search strategy incorporated the following keywords: “brachial artery aneurysm”, “true brachial artery aneurysm”, “peripheral aneurysm”, “upper limb aneurysm”, and “upper extremity aneurysm”. The retrieved records were subsequently screened for relevance to the topic of the study. A cross-reference was carried out to retrieve relevant studies. We included only the idiopathic true aneurysms. BAAs in the context of congenital syndromes, trauma, infection, connective tissue disorders, arteriovenous malformations, immunosuppression, and a history of arteriovenous vascular access were excluded. Brachial artery pseudoaneurysms, usually caused by trauma and the increasing use of invasive procedures (e.g., arterial lines, dialysis access, cardiac catheterizations), were also excluded.

## 5. Results

Given the heterogeneity of reporting across published cases, aggregated percentages are reported using variable denominators corresponding to the number of cases in which each variable was available.

Regarding sex predominance, 22 (56%) were male, 17 (44%) female, and in 5 cases the sex could not be extracted in the relevant cases series. Regarding the side affected, 16 (43%) were located on the right side, 21 (57%) on the left side, and in 1 patient, they were bilateral. The mean age was 34.3 years. In 4 cases, age could not be extracted in the relevant case series. Regarding symptomatology, 21 (58%) patients were symptomatic, 15 (42%) were asymptomatic, in 1 patient with bilateral aneurysms,1 was symptomatic and the other asymptomatic, and in 7 cases the symptomatology could not be defined. In symptomatic pts the most common presentation was painful swelling ± palpable mass. Eight pts presented with acute ischemia where Fogarty embolectomy was performed in addition to standard repair [2,17,31,32,33,34,35,36]. Among them, 2 pts had aneurysm thrombosis, 5 had distal embolism, and 1 had aneurysm thrombosis plus distal embolism. Three experienced medial nerve compression [10,11,29]. One presented with digital gangrene [29]. Among the cases in which aneurysm size was reported, the mean axial diameter was approximately 2.9 cm, and the maximum axial diameter was estimated at 3.2 cm. In 7 cases, the size could not be extracted. In 20 cases, the duration of symptoms before seeking care was reported, and the mean time elapsed was 15.5 months. The principal symptom in asymptomatic patients was the feeling of a pulsatile mass and/or swelling. In 38 cases where the imaging method was reported, CDU alone was used in 10 pts (26%), CTA alone in 11 pts (29%), CDU plus CTA in 7 (18%), CDU plus MRA in 6 (16%) pts, DSA in 1 pt and in another one the aneurysm was incidentally found during coronary angiography [37]. Regarding treatment, aneurysm excision was performed in all but 3 pts (In 1 pt, simple ligation was performed, in the 2nd embolization of the sac after ligation and bypass procedure, and in the 3rd, where the aneurysm was incidentally found during coronary angiography, conservative management was decided). Restoration of arterial continuity was achieved with interposition grafting in most cases, 31/41 (76%). Reversed GSV was the graft of choice used in 22/31 (71%). Reversed BV was used in 5/31 (16%), and reversed CV in 1/31 pts (0.3%) and PTFE in 2/31 pts (0.6%). In one pt a biological graft (Omniflow) was used [17]. In 6/41 pts (15%), a primary end-to-end anastomosis was achieved. In 1 pt where the sac was embolized after aneurysm ligation, the GSV was not interposed but placed as a bypass graft [38]. In 1 true BAA with saccular morphology, lateral aneurysmorrhaphy was performed. In 1 pt, two different GSV grafts were used, and in 1 pt, a custom GSV bifurcated graft was constructed for repair [2,39]. In 1 child, the internal iliac artery was used as a graft [40]. In 1 case, the technique was not reported, and in 2 cases, the kind of interposed graft was not reported. The mean follow-up time was 15.4 months in 16 cases where it was reported. No complications were reported at the mid-term.

Our literature review (summarized in Table 2) underscores the rarity yet clinical significance of BAA diagnosis and repair, since only 32 case reports and 5 small case series with 44 relevant patients were retrieved in the published English literature [40,41,42,43,44,45,46,47,48,49,50,51,52,53,54,55,56,57,58,59].

Our patients were all male with mean age 46.3 years. In one pt the aneurysm was on the right side and in the other two on the left. All were asymptomatic with a mean aneurysm diameter of 3.8 cm. In contrast to the widespread use of GSV we preferred the BV as it was of adequate size. A concise overview of the main aggregated findings from the scoping review is provided in Table 3, highlighting key clinical characteristics, management strategies, and outcome patterns reported in the literature.

### 5.1. Incidence

Arterial aneurysms of extremities account for 18% of all arterial aneurysms, with popliteal and common femoral the most common [11]. Upper extremity aneurysms are less common compared to lower extremity aneurysms and account for less than 1% of all peripheral aneurysms [2]. Although BAAs account for 0.5%, true BAAs account for 0.17% of all peripheral aneurysms and usually manifest as a painless mass [2].

### 5.2. Pathophysiology

Atherosclerotic risk factors (hypertension, hyperlipidemia, smoking, and age) may exist in patients with idiopathic true BAAs [35]. Smoking was apparent in 25% and hypertension in 33% of patients in one reported series [40]. Generally, histopathologic examination reveals medial degeneration, fibrosis, and disruption of the elastic laminae [12]. In most reports, true BAAs are usually isolated and rarely associated with other aneurysms. In our review, only in three cases were they associated with axillary aneurysms at the time of diagnosis [17]. However, in one report, they coincide with other aneurysms at a rate of 8% [40]. Genetic testing is suggested [41]. It has revealed variations in MYH7 and COL7A1 genes, which affect protein structure in myosin subunits in myocardium and type 7 collagen, respectively. However, the relationship of these specific heterozygous mutations with aneurysms is unknown, and further research is needed [41].

Traumatic aneurysms are mainly saccular and present as pseudoaneurysms. Sometimes, occult repetitive chronic trauma may be implicated in the pathophysiological process of true BAAs [18,39]. The use of crutches may cause degenerative lesions to the axillary artery due to chronic repetitive trauma and predispose to aneurysm formation [42]. This pattern can also cause aneurysm of the brachial artery [43]. Handling of the fishing ropes by fishermen has also been implicated in the pathophysiology of true BAAs [3,39]. In all these cases, chronic repetitive occult trauma leads to compression of the arterial wall with subsequent contusion of the media. This results in weakness of the arterial wall and fusiform dilatation (not saccular) [40].

AVF-related brachial artery aneurysms can be present while AVF is working, after AVF ligation, and after transplantation [7,44]. In the presence of an AVF, shear stress created by fistula flow is implicated in aneurysm formation [38]. AVF-related are the most common BAAs [2]. The time interval between AVF creation and aneurysmal diagnosis was about 20.6 years in one report [45]. As mentioned above, these BAAs are excluded from this review.

### 5.3. Clinical Symptoms

When symptomatic, local pain or tenderness is the predominant symptom, along with signs of a pulsatile mass. At the time of diagnosis, atherosclerotic BAAs tend to be larger and are palpable in two-thirds of patients with an accompanying audible bruit in some cases [17]. Complications include median nerve compression and paresthesia, venous compression leading to insufficiency and arm edema, due to a mass effect [24]. Sometimes, compressive effects may lead to impaired limb function [24]. Acute arm ischemia may result from thrombosis or distal embolization [4,17,24,31]. A patient may have a ‘known’ mass in his arm for many years, which suddenly may become symptomatic, causing acute limb ischemia due to aneurysm thrombosis, dislodgement of luminal thrombus, and obstruction of the outflow vessel, embolism in distal arteries, or digital micro-embolism [2,11,17,32,34,39,46,47]. Chronic microembolization may lead to permanent occlusion of distal arm arteries with digital tissue loss, ulceration, and gangrene [17]. These obstructions may not be amenable to Fogarty embolectomy [33]. Consequently, physical examination in patients with neurovascular complaints of the upper extremities is very important [2].

### 5.4. Diagnosis

Diagnosis is based on clinical examination supplemented with color duplex ultrasonography (CDU) imaging, which is usually the first examination performed. It is preferred as it is quick, economical, and delineates the morphology of the aneurysm, but sometimes the findings may be misleading [40]. Computed tomography angiography (CTA) and magnetic resonance angiography (MRA) are confirmatory exams guiding the surgical plan. MRA is a valuable diagnostic method in the pediatric population due to its high image resolution and lack of radiation exposure [14,48]. BAAs should be differentiated from pseudoaneurysms, hematomas, vascular malformations, ganglion or synovial cysts, abscesses, neural tumors, muscular fibromas, and other upper extremity lesions [1,20,49]. Whole-body arterial imaging is needed to exclude aneurysms at other locations [4,40].

### 5.5. Treatment

The treatment options of BAAs have been generally extrapolated from case reports and case series [1,50]. Operative repair still remains the ‘gold standard’ for upper extremity arterial aneurysms, since the first report by Griffith in 1897 [40,46]. Brachial plexus block anesthesia can be used as an alternative to general anesthesia [49,51]. Local anesthesia is not an option for children but is an alternative for older patients [49]. Acute limb ischemia needs urgent treatment to avoid irreversible hand ischemia, acute compartment syndrome, gangrene, and amputation [31]. A preoperative ultrasound mapping of the upper extremities’ superficial veins (mainly basilic and cephalic) and the great saphenous lower extremities is always useful for harvesting. In the absence of adequate-sized veins or if they have been harvested (e.g., for CABG), a prosthetic graft may be used [1,17]. The choice of operative technique is based on the length of the arterial defect [24]. If the artery is elongated, aneurysm excision along with end-to-end anastomosis may be achieved [4,11,22,52]. Otherwise, the defect is bridged with graft interposition. If total aneurysm excision is not possible because the aneurysm’s bottom is strictly connected to nearby structures, this part could be left in place. Primary patency of interposition grafting was 77% at 2.4 years in one report [4]. However, vein grafts have better long-term patency [2].

In one report, a bifurcated saphenous vein graft was used to revascularize both radial and ulnar arteries, as aneurysmatic disease was extended to one of them [39]. In another report, two different GSV bypass grafts were used to revascularize the radial and ulnar arteries independently [2]. Thus, ligation of the radial or ulnar artery was avoided [31]. In our case 2, we proceeded to ligation of the ulnar artery and revascularized the radial artery. Conversely, Tadayon N et al. performed ligation of the radial artery and revascularized the ulnar artery [31]. In case of embolic occlusions of the distal run-off arteries, an embolectomy should be performed before completion of the distal anastomosis [17]. In one case, postoperative prosthetic graft thrombosis necessitated restoration with an autogenous venous conduit [24]. In autogenous venous grafts, dilatation in the long term is a rare issue [24]. Aneurysmal degeneration of the distal artery 3-years after successful repair of a BAA has been reported. Re-operation was successful [27]. In one report, simple ligation was performed in 5 out of 10 aneurysms [31]. In another report, palpable distal pulses were noted in a 5-year-old girl, 6months after simple ligation [53]. Factors that permitted this approach were the location of the aneurysm, adequate back bleeding, normal capillary refill after arterial occlusion, and difficulty of primary repair [31,34].

In children, 7-0 to 9-0 interrupted nylon sutures are mainly used for anastomosis [54]. In an 18-month-old girl with a small BAA and asymptomatic outflow vessel obstruction, although aneurysm ligation would be an option, the authors proceeded to venous bypass, concerned about extremity growth on the first occasion [54]. Limb growth disturbances have been described in pediatric iliac arterial aneurysms, improving after surgical repair [55,56]. In infants, there is a surgical challenge regarding the very small diameter of the brachial artery (1–2.5 mm) [55,57,58]. The precise timing for operation in asymptomatic children is controversial. Some suggest an observation strategy aiming at a subsequent surgical repair at an older age [29,59]. This approach perhaps has a role in small asymptomatic aneurysms without luminal thrombus [46,59]. Others suggest a quick operation after the diagnosis to avoid limb ischemia [14,60]. In a series of Nurmeev I et al., with 5 children with a mean age of 3.3 years, all were operated on, and no one was elected for regular observation [24].

Definitely, early surgery should be recommended at all ages for moderately sized to large aneurysms that increased in size recently, formed luminal thrombus, or caused distal neurovascular compromise [46]. An increase in diameter above 2 times the normal size of the artery is the general threshold for aneurysm repair [61]. A threshold of 2.5 cm with the presence of intraluminal thrombus was the indication for repair in asymptomatic patients in a recent report [4,36]. Although it is not impossible to predict which asymptomatic aneurysms are likely to develop complications, it is reported that the complication rate is 33% in asymptomatic patients in a six-year period [2,40,60]. Conservative management was safe for asymptomatic aneurysms smaller than 2.5 cm, in one recent report [4]. Conversely, others suggest operative repair in all BAAs based on the low morbidity associated with surgery [40].

Endovascular repair with covered stents has emerged as an alternative treatment modality and has been successfully applied in subclavian and axillary artery aneurysms, as well as in non-idiopathic brachial artery aneurysms [62,63,64,65,66,67,68,69]. Endovascular techniques have also been reported for peripheral arterial aneurysms; however, a higher incidence of stent thrombosis has been observed in some series, raising concerns regarding durability in mobile arterial segments [63,65,70].

In the specific context of brachial artery aneurysms, several anatomical and biomechanical limitations may restrict the applicability of endovascular exclusion. These include arterial tortuosity, diameter mismatch between proximal and distal landing zones, multilobulated aneurysm morphology, presence of intraluminal thrombus, and the marked mobility of the brachial artery, particularly across the elbow joint. In addition, endovascular exclusion does not address compressive symptoms, such as median nerve compression, which may persist despite successful aneurysm exclusion [11]. Stent fracture related to repetitive arm flexion has also been reported, and as a result, many patients are not anatomically suitable candidates for endovascular repair [17].

In pediatric patients, further concerns include stent migration and compromised long-term patency associated with limb growth, limiting the applicability of endovascular treatment in this population [57]. In contrast, open surgical exposure of the brachial artery is relatively straightforward, and surgical repair has been associated with low perioperative morbidity and mortality and excellent long-term patency, particularly when autologous venous conduits are used [17].

Importantly, no cases of endovascular repair for idiopathic true brachial artery aneurysms have been reported in the available literature. This absence of reported experience likely reflects both the rarity of the disease and the aforementioned anatomical constraints rather than definitive evidence against the technique. Consequently, while endovascular repair may represent a valuable option in selected clinical scenarios—such as emergency settings, patients with significant comorbidities, or non-idiopathic aneurysms, including traumatic or iatrogenic pseudoaneurysms—current evidence does not support its routine elective use in idiopathic true brachial artery aneurysms, for which open surgical repair remains the most established treatment option. Mid- and long-term patency data for endovascular repair in this anatomical location remain limited [4]. Long-term follow-up is generally recommended, as aneurysms at other arterial sites may develop many years after initial presentation [29,57,59,71].

## 6. Limitations

This study has several limitations that should be acknowledged. First, due to the retrospective nature of the case series and the long study period, formal outpatient follow-up data were not available for the included patients. Although early postoperative outcomes—including restoration of distal pulses and uneventful in-hospital recovery—were documented in all three cases, duplex ultrasonography and standardized short-, mid-, or long-term follow-up could not be retrieved. Attempts to contact patients using recorded telephone details were unsuccessful due to outdated contact information.

Second, the small sample size reflects the extreme rarity of idiopathic true brachial artery aneurysms and limits the ability to draw generalized conclusions regarding long-term patency or comparative effectiveness of treatment strategies.

Third, the extended inclusion period may have introduced heterogeneity related to diagnostic modalities, perioperative management, and documentation practices over time. The exceptionally long inclusion period reflects the extreme rarity of idiopathic true brachial artery aneurysms. However, this inevitably introduces heterogeneity related to evolving diagnostic imaging modalities, perioperative management strategies, and documentation standards, particularly in earlier cases. Consequently, the institutional case series is intended to serve a descriptive and illustrative purpose, highlighting clinical presentation, operative principles, and pathological confirmation, rather than to enable comparative outcome assessment or temporal trend analysis.

Moreover, the absence of structured follow-up data reflects both the retrospective design and the extended study period, during which standardized follow-up protocols and electronic documentation were not uniformly implemented. Finally, the exclusion of brachial artery aneurysms associated with connective tissue disorders, trauma, or arteriovenous fistulas—although deliberate to preserve pathophysiological homogeneity—limits the applicability of our findings to idiopathic cases only.

## 7. Conclusions

Idiopathic true brachial artery aneurysms are exceedingly rare and may lead to limb-threatening ischemic complications if left untreated. The most common clinical finding is a palpable mass in the upper arm, underlining the importance of careful physical examination in patients with atypical upper-extremity symptoms. In our institutional case series, open surgical repair with aneurysm excision and revascularization resulted in favorable early postoperative outcomes, including preserved distal perfusion and uneventful in-hospital recovery. Due to the absence of structured long-term follow-up, no conclusions regarding durability can be drawn from the institutional cases alone.

However, evidence from the published literature, summarized in this scoping review, suggests that open surgical repair—most commonly using autologous venous conduits—has been associated with satisfactory mid- and long-term outcomes. Taken together, these findings support open surgical repair as the most established treatment option for idiopathic true brachial artery aneurysms, while highlighting the need for structured follow-up and further data to better define long-term durability.

## Figures and Tables

**Table 1:** Demographics, clinical, and operative data of our patients.

Patient ID/Year	Age/Sex	Symptoms/ Signs	Side/ Location	Size (cm)	Repair	Follow-Up
1/ 2001	28/M	No/ Palpable mass	R/Distal third of BA, extending to ulnar artery	3.5	Aneurysm excision & reversed BV interposition between BA & RA, UA ligation	NA
2/ 2007	46/M	No/ Palpable mass	L/ Distal half of BA	4	Aneurysm excision & reversed BV interposition	NA
3/ 2015	65/M	No/ Palpable mass	L/ Two proximal thirds of BA	4	Aneurysm excision & reversed BV interposition	NA

Abbreviations: ID: identification number, M: male, R: right, L: left, BA: brachial artery, BV: basilic vein, RA: radial artery, UA: ulnar artery, NA: not available.

**Table 2:** All reported cases of true idiopathic brachial artery aneurysms. Demographics, clinical and operative data are presented.

ID	Author/Ref	Journal/ Year	Age/ Sex	Symptoms/Side	Signs/ Size (cm)	Time	DI	Treatment/ Fu
1	Berhanu DL et al. [20]	Int J Surg Case Rep/2025	3 y/ F	No/ L	Painless swelling/1.7 × 1.6	2 w	CDU, CTA	AE & Interposition grafting with GSV graft/ NR
2	Ali AA et al. [1]	Int J Surg Case Rep/2025	28 y/ F	Yes/ L	Painful swelling/NR	2 y	CDU	AE & Interposition grafting with PTFE graft 8 mm/ 6 m
3	Zhang L et al. [5]	Eur J Vasc Endovasc Surg/ 2025	14 y/ M	No/ L	Pulsatile mass/ 3 × 3	NR	CTA	AE & Interposition grafting with BV graft/ 6 y
4	Liao Z et al. [22]	Asian J Surg/ 2024	78 y/ F	No/ L	Pulsating mass/ 3.4 × 2.2 × 3.5	2 y	CTA	AE & Interposition grafting with PTFE graft/ 5 y
5	Lee CYV et al. [48]	Vasc Endovasc Surg/ 2024	9 m/ NR	No/ L	Swelling/ 2 × 2	2 m	CDU, MRA	AE & Interposition grafting with CV vein graft/ NR
6	Gonzalez-Urquijo M et al. [38]	Vasc Endovascular Surg/2022	65 y/ F	Yes/ L	Pain, pulsatile mass/ 2 × 6	NR	CDU, MRA	Bypass with GSV graft & sac embolization with Gelita-spon/ NR
7	Bautista-Sánchez J et al. [50]	Ann Vasc Surg/ 2022	67 y/ F	No/ L	pulsatile mass/ NR	NR	NR	AE & Interposition grafting with GSV graft/ NR
8	Tadayon N et al. [31]	J Cardio-vasc Thorac Res/2020	66 y/ F	Yes/ R	Aneurysm thrombosis & distal embolism, AI/ 2 × 2.5 × 3	12 h	CTA	AE & Interposition grafting with GSV graft, ligation of RA/ 6 m
9	Shaban Y et al. [2]	Ann Med Surg (Lond)/2020	83 y/ M	Yes/ R	Pain, swelling, pulsatile mass, AI/ 0.9	1 w	CDU, CTA	AE & Interposition grafting with GSV (2 grafts)/ 6 m
10	Nurmeev I et al. [24]	Case Rep Med/ 2020	NR	NR	NR	NR	CDU	AE & Interposition grafting/ 1–3 y
11	Taghi H et al. [11]	J Med Vasc/ 2020	70 y/ F	Yes/ R	Paresthesia, swelling, nerve compression/ 2.6 × 3.5	3 y	CDU, CTA	AE & End-to-end anastomosis/ NR
12	Senarslan DA et al. [17]	Ann Vasc Surg/ 2019	27 y/ M	Yes/ L	Pain, numbness, embolism, AI/ 3 aneurysms: 3 × 2.8, 4.7 × 4.4, 2.4 × 2.1	NR	CTA	Interposition grafting with GSV graft/ NR
13			81 y/ F	Yes/ L	Digital gangrene/ 5 × 10	NR	CTA	AE & Interposition grafting with GSV graft (proximal anastomosis end-to-side due to increased axillary diameter)/ 12 m
14			78 y/ M	Yes/ L	Pain, cyanosis, numbness/ 3 × 10	NR	CTA	AE & Interposition grafting with biological graft Omniflow/ 6 m
15	Pradhananga A et al. [32]	J Nepal Med Assoc/2017	59 y/ M	Yes/ L	Pain, AI/ NR	8 h	CDU	AE & Interposition grafting with GSV graft/ NR
16	Ghazanfar A et al. [46]	BMJ Case Rep/ 2016	2 y/ M	No/ R	Swelling/ 4 × 3	NR	CDU, CTA	AE & Interposition grafting with GSV graft/ NR
17	Yuan Y et al. [47]	Eur J Vasc Endovasc Surg/ 2016	38 y/ M	Yes/ R	Painful pulsatile mass/ 3.5 × 4	NR	CTA	AE & Interposition grafting with GSV graft/ NR
18	Ben Mrad M et al. [39]	Ann Vasc Surg/ 2016	40 y/ M	Yes/ L	Painfull pulsatile mass/3.7 × 4.2 × 6 cm	NR	CTA	AE & Interposition grafting with GSV bifurcated graft/ 1 y
19	Greenberg JI et al. [54]	J Vasc Surg/ 2012	18 m/F	Yes/ L	Arm swelling/ 1.2	NR	CTA	AE & Interposition grafting with GSV graft/ NR
20	A Fakhree MB et al. [33]	J Cardiovasc Thorac Res/2012	67 y/ M	Yes/ R	Aneurysm thrombosis, AI/ 2	1 w	CTA	AE & Interposition grafting with GSV graft/ 1 m
21	Alagaratnam S et al. [10]	Ann R Coll Surg Engl/2011	64 y/ F	Yes/ L	Swelling, paresthesia nerve compression/ 3 × 5 cm	2 m	CDU, CTA	AE & Interposition grafting with GSV graft/ NR
22	Tetik O et al. [49]	Tex Heart Inst J/ 2010	50 y/ F	Yes/ R	Swollen pulsatile mass/ 4 × 2.5	NR	DSA	AE & Interposition grafting with GSV graft/ NR
23	Hudorović N et al. [12]	Wien Klin Wochenschr/ 2010	77 y/ F	No/ L	Swelling/ 5 × 4	2 y	CDU, CTA	AE & Interposition grafting with GSV graft/ NR
24	Bahcivan M et al. [14]	Interact Cardiovasc Thorac Surg/ 2009	9 m/ M	No/ L	Pulsatile mass/ 3.5 × 3.2	1 m	CDU, MRA	AE & End to end anastomosis/ NR
25	Pagès ON et al. [57]	Pediatr Surg Int/2008	3 m/ M	NR	NR/ 0.6	NR	CDU/MRA	AE & End to end anastomosis/ NR
26			1 y/M	NR	NR /3	NR	CDU/MRA	AE & Interposition grafting with BV graft/ NR
27			13 m/M	NR	NR	NR	CDU/MRA	AE & Interposition grafting with BV graft/ NR
28	Gray RJ et al. [40]	J Vasc Surg/ 1998	NR	NR	Aneurysm thrombosis/ 5.3	NR	NR	AE & revascularization with GSV graft/ NR
29			NR	NR	Symptomatic mass/ 3.8	10 y	NR	AE & revascularization with internal iliac artery graft/ NR
30	Gangopadhyay N et al. [29]	Plast Reconstr Surg Glob Open/2016	7 m/ M	Yes/ L	Impaired MN function/ 2 × 2.4	10 d	CDU, MRA	NR/ 1 m
31	Jones TR et al. [59]	J Vasc Surg/ 1988	9 m/ M	No/ L	Pulsatile mass/ 0.6 × 1	NR	CDU	AE & End-to-end anastomosis/ NR
32	Jardine-Brown CP et al. [34]	Proc R Soc Med/ 1972	78 y/ M	Yes/ R	AI, pain, cyanosis/ 7 × 5	10 d	NR	AL & GSV bypass & homolateral cervical sympathectomy/ NR
33	Fann JI et al. [36]	J Pediatr Surg/ 1994	3 y/ M	Yes/L No/R	L: AI/ 1.8 × 3.5 R: Pulsatile mass/ 1.1 × 2.6	NR	CDU	L&R: AE & Interposition grafting with GSV graft/ NR
34	Hirji SA et al. [16]	Journal of Pediatr Surg Case Rep/ 2017	9 m/ F	No/ L	Pulsatile mass/ 1.7 × 1	2 w	CDU	AE & Interposition grafting with BV graft/ NR
35			3 y/ F	No/ R	Pulsatile mass/ 3 × 0.9	Few w	CDU, MRA	AE & Interposition grafting with GSV graft/ NR
36	Mowafy KA et al. [58]	Int J Angiol Vasc Surg/2020	6 m/ M	Yes/ R	Tender swelling, impaired, painful mobility/ 5.5 × 1.8	1 m	CDU	AE & Interposition grafting with BV graft/ NR
37	Vasavada A et al. [37]	BMJ Case Rep/ 2015	58 y/ M	No/ R	Incidental finding during CA/ NR	NR	CA	Conservative management/ 3 m
38	Heydari F et al. [35]	Emerg (Tehran)/2015	52 y/ M	Yes/ R	AI/2.5 × 2.2	NR	CDU, CTA	AE & Interposition grafting with GSV graft, embolectomy
39	Raja N et al. [51]	Indian J of Vasc and Endo-vasc Surg/ 2017	53 y/ M	Yes/ R	Pulsatile mass/ 4	NR	CDU	AE & Interposition grafting with GSV graft, embolectomy/ NR
40	Edavalapati S et al. [41]	Am Surg/2022	28 y/ F	No/ L	Pulsatile mass/ 2	NR	CDU, MRI	AE & End-to-end anastomosis/6 m
41	Kaikaus J et al. [15]	Annals of Vasc Surg—Brief Rep and Innov/ 2025	6 m/ M	No/ L	Pulsatile mass/ 2	NR	CTA	AE & Lateral aneurysmorraphy/ NR
42	Ghazi MA et al. [52]	Japan Med Assoc J/ 2006	27 y/ F	No/ R	Painless swelling/ 3.2 ×2	6 y	CDU	AE & End-to-end anastomosis/ 2 m
43	Parvin SD et al. [53]	Eur J Vasc Surg/ 1987	5 y/ F	Yes/ R	Pulsatile swelling/3 × 2	8 w	NR	AL/ 6 m

Abbreviations: ID: identification number, Ref: reference, DI: diagnostic Imaging, Fu: follow-up, M: male, F: female, R: right, L: left, CDU: color duplex ultrasonography, CTA: computed tomography angiography, MRA: magnetic resonance angiography, CA: coronary angiography, y: years, m: months, w: weeks, d:days, PTFE: polytetrafluoroethylene, AI: acute ischemia, AE: aneurysm excision, AL: aneurysm ligation, BV: basilic vein, CV: cephalic vein, GSV: great saphenous vein, MN: median nerve, NR: not reported. (‘Time’ refers to the duration of symptoms/signs before presentation).

**Table 3:** Overview of key findings from the scoping review of idiopathic true brachial artery aneurysms.

Domain	Key Observations from the Literature
Patient characteristics	Predominantly adults; both sexes affected; idiopathic etiology without trauma, infection, or connective tissue disease
Clinical presentation	Most commonly a palpable upper-arm mass; may be associated with pain, tenderness, or local compression symptoms; ischemic manifestations reported less frequently; some cases detected incidentally
Aneurysm morphology and location	Typically, fusiform true aneurysms; most often involving the mid-to-distal brachial artery, occasionally extending to the bifurcation
Diagnostic modalities	Duplex ultrasonography as first-line imaging; computed tomography angiography frequently used for anatomical delineation and operative planning
Treatment strategy	Predominantly open surgical repair; conservative management reported rarely and mainly in asymptomatic or high-risk patients
Surgical techniques	Aneurysm excision with arterial reconstruction most commonly performed; end-to-end anastomosis feasible in selected cases
Conduit choice	Not reported for idiopathic true brachial artery aneurysms; endovascular approaches described mainly for non-idiopathic aneurysms or pseudoaneurysms
Reported outcomes	Early postoperative outcomes generally favorable; mid- and long-term outcomes derived from literature reports rather than institutional follow-up
Follow-up considerations	Long-term surveillance recommended due to potential development of aneurysms at other arterial sites

## Data Availability

Research data are stored in an institutional repository and will be shared upon reasonable request to the corresponding author.

## Supplementary Materials

The following supporting information can be downloaded at: https://www.mdpi.com/article/10.3390/jcm15010295/s1, Table S1: PRISMA Extension for Scoping Reviews (PRISMA-ScR): Checklist of items to include in reports of scoping reviews.

## References

[1] Ali A.A., Hussein A.M., Abdi H.K., Siyad A.A.A., Keilie A.M.W., Ahmed F.M. (2025). Spontaneous true brachial artery aneurysm: A case report from Somalia. Int. J. Surg. Case Rep..

[2] Shaban Y., Elkbuli A., Geraghty F., Boneva D., McKenney M., De La Portilla J. (2020). True brachial artery aneurysm: A case report and review of literature. Ann. Med. Surg..

[3] Igari K., Kudo T., Toyofuku T., Jibiki M., Inoue Y. (2013). Surgical treatment of aneurysms in the upper limbs. Ann. Vasc. Dis..

[4] Zheng A., Sen I., De Martino R., Erben Y., Davila V., Ciresi D., Beckermann J., Carmody T., Tallarita T. (2025). Presentation, treatment, and outcomes of brachial artery aneurysms. J. Vasc. Surg..

[5] Zhang L., Li X. (2025). True Brachial Artery Aneurysm in an Adolescent. Eur. J. Vasc. Endovasc. Surg..

[6] Guntani A., Takeshita M., Yasunaga C., Nakayama K., Mii S., Komori K. (2025). Inflammatory brachial artery aneurysm with amyloidosis due to nontuberculous mycobacterial infection: A case report. J. Vasc. Surg. Cases Innov. Tech..

[7] Ouhmich M., Banana Y., Anane O., Rezziki A., Benzirar A., El Mahi O. (2025). Brachial Artery Aneurysm After Fistula Ligation in a Hemodialysis Patient: A Case Report. Cureus.

[8] Murugesan P., Yesuvadiyan J.P., Selvaraj K., Pattabi S. (2025). A Time Bomb in the Arm: Rare Delayed Presentation of a Post-traumatic True Brachial Artery Aneurysm. Cureus.

[9] Hammad Alam S.M., Moin S., Gilani R., Jawad N., Abbas K., Ellahi A. (2024). True brachial artery aneurysm: A systematic review. J. Pak. Med. Assoc..

[10] Alagaratnam S., Choong A., Lau T., Munro M., Loh A. (2011). Swelling in the upper arm: The presentation and management of an isolated brachial artery aneurysm. Ann. R. Coll. Surg. Engl..

[11] Taghi H., Zahdi O., Hormat-Allah M., Bakkali T., El Bhali H., Sefiani Y., El Mesnaoui A., Lekehal B. (2020). Idiopathic true isolated aneurysm of the brachial artery. J. Med. Vasc..

[12] Hudorović N., Lovričević I., Franjić D.B., Brkić P., Tomas D. (2010). True aneurysm of brachial artery. Wien. Klin. Wochenschr.

[13] Ramakrishna P., Mahapatra S., Rajesh R. (2013). True Aneurysm of the Proximal Brachial Artery. Aorta.

[14] Bahcivan M., Yuksel A. (2009). Idiopathic true brachial artery aneurysm in a nine-month infant. Interact. Cardiovasc. Thorac. Surg..

[15] Kaikaus J., Moseley M.D., Dwivedi A.J., Sigdel A. (2022). A Large True Brachial Artery Aneurysm in an Infant. Am. Surg..

[16] Hirji S.A., Knell J.K., Kim H.B., Fishman S.J., Taghinia A. (2017). Spontaneous isolated true aneurysms of the brachial artery in children. J. Pediatr. Surg. Case Rep..

[17] Senarslan D.A., Yildirim F., Tetik O. (2019). Three Cases of Large-Diameter True Brachial and Axillary Artery Aneurysm and a Review of the Literature. Ann. Vasc. Surg..

[18] Clark E.T., Mass D.P., Bassiouny H.S., Zarins C.K., Gewertz B.L. (1991). True aneurysmal disease in the hand and upper extremity. Ann. Vasc. Surg..

[19] Wanhainen A., Van Herzeele I., Bastos Goncalves F., Bellmunt Montoya S., Berard X., Boyle J.R., D’Oria M., Prendes C.F., Karkos C.D., Kazimierczak A. (2024). Editor’s Choice—European Society for Vascular Surgery (ESVS) 2024 Clinical Practice Guidelines on the Management of Abdominal Aorto-Iliac Artery Aneurysms. Eur. J. Vasc. Endovasc. Surg..

[20] Berhanu D.L., Mendere D.B., Chala Z.G., Bekele F.A. (2025). True brachial artery aneurysm in a 3-year-old: A case report. Int. J. Surg. Case Rep..

[21] Sowdagar S., Jha A., Vasanth P., Mathew J. (2025). A Clinical Case Report of Deficiency of Adenosine Deaminase 2 Syndrome (DADA 2) Presenting as a Brachial Artery Aneurysm. Mediterr. J. Rheumatol..

[22] Liao Z., Zhang Y. (2024). Treatment of idiopathic true giant brachial aneurysm using artificial vascular graft: A case report. Asian J. Surg..

[23] Chimada B.Y., Hachiro K., Takashima N., Suzuki T. (2023). Successful revascularization using a saphenous vein for a ruptured brachial artery aneurysm in a patient with neurofibromatosis type I. J. Vasc. Surg. Cases Innov. Tech..

[24] Nurmeev I., Osipov D., Okoye B. (2020). Aneurysm of Upper Limb Arteries in Children: Report of Five Cases. Case Rep. Med..

[25] Sarkar R., Coran A.G., Cilley R.E., Lindenauer S.M., Stanley J.C. (1991). Arterial aneurysms in children: Clinicopathologic classification. J. Vasc. Surg..

[26] Davis F.M., Eliason J.L., Ganesh S.K., Blatt N.B., Stanley J.C., Coleman D.M. (2016). Pediatric nonaortic arterial aneurysms. J. Vasc. Surg..

[27] Ko S., Han I.Y., Cho K.H., Lee Y.H., Park K.T., Kang M.S. (2011). Recurrent true brachial artery aneurysm. Korean J. Thorac. Cardiovasc. Surg..

[28] Godwin S.C., Shawker T., Chang B., Kaler S.G. (2006). Brachial artery aneurysms in Menkes disease. J. Pediatr..

[29] Gangopadhyay N., Chong T., Chhabra A., Sammer D.M. (2016). Brachial Artery Aneurysm in a 7-Month-Old Infant: Case Report and Literature Review. Plast. Reconstr. Surg. Glob. Open.

[30] Dayanır O.B., Silistreli E.E. (2024). [MEP-02] A Buerger’s Patient with Brachial Artery Aneurysm. Turk. Gogus. Kalp. Damar. Cerrahisi. Derg..

[31] Tadayon N., Zarrintan S., Kalantar-Motamedi S.M.R. (2020). Acute right upper extremity ischemia resulting from true aneurysm of right brachial artery: A case report. J. Cardiovasc. Thorac. Res..

[32] Pradhananga A., Chao X. (2017). True Brachial Artery Aneurysm Presenting as a Non-Pulsatile Mass. JNMA J. Nepal. Med. Assoc..

[33] Fakhree M.B.A., Azhough R., Hafez Quran F. (2012). A case of true brachial artery aneurysm in an elderly male. J. Cardiovasc. Thorac. Res..

[34] Jardine-Brown C.P. (1972). Right brachial artery arteriosclerotic aneurysm with distal thromboembolic phenomena. Proc. R. Soc. Med..

[35] Heydari F., Taheri M., Esmailian M. (2015). Brachial Artery Aneurysm as a Limb Threatening Condition: A Case Report. Emergency.

[36] Fann J.I., Wyatt J., Frazier R.L., Cahill J.L. (1994). Symptomatic brachial artery aneurysm in a child. J. Pediatr. Surg..

[37] Vasavada A., Agrawal N., Parekh P., Vinchurkar M. (2015). Incidentally detected large idiopathic brachial artery aneurysm: A potentially life-threatening discovery. BMJ Case Rep..

[38] Gonzalez-Urquijo M., Marine L., Vargas J.F., Valdes F., Mertens R., Bergoeing M., Torrealba J. (2022). True Idiopathic Brachial Artery Aneurysm Treated with a Saphenous Vein Graft. Vasc. Endovascular. Surg..

[39] Ben Mrad M., Neifer C., Ghedira F., Ghorbel N., Denguir R., Khayati A. (2016). A True Distal Brachial Artery Aneurysm Treated with a Bifurcated Saphenous Vein Graft. Ann. Vasc. Surg..

[40] Gray R.J., Stone W.M., Fowl R.J., Cherry K.J., Bower T.C. (1998). Management of true aneurysms distal to the axillary artery. J. Vasc. Surg..

[41] Edavalapati S., Hamilton C., Curtiss S. (2025). Idiopathic brachial true aneurysm in 28-year-old female. Ann. Vasc. Surg.—Brief Rep. Innov..

[42] Davidovic L.B., Kostic D.M., Jakovljevic N.S., Kuzmanovic I.L., Simic T.M. (2003). Vascular thoracic outlet syndrome. World J. Surg..

[43] Konishi T., Ohki S., Saito T., Misawa Y. (2009). Crutch-induced bilateral brachial artery aneurysms. Interact. Cardiovasc. Thorac. Surg..

[44] Chemla E., Nortley M., Morsy M. (2010). Brachial artery aneurysms associated with arteriovenous access for hemodialysis. Semin. Dial..

[45] Fendri J., Palcau L., Cameliere L., Coffin O., Felisaz A., Gouicem D., Dufranc J., Laneelle D., Berger L. (2017). True Brachial Artery Aneurysm after Arteriovenous Fistula for Hemodialysis: Five Cases and Literature Review. Ann. Vasc. Surg..

[46] Ghazanfar A., Asghar A., Khan N.U., Abdullah S. (2016). True brachial artery aneurysm in a child aged 2 years. BMJ Case Rep..

[47] Yuan Y., Lu H.J. (2016). True Brachial Artery Aneurysm. Eur. J. Vasc. Endovasc. Surg..

[48] Lee C.Y.V., Natalwala I., Tahir N., Bains R.D. (2024). A Rare Case of Brachial Artery Aneurysm in a 9-Month-Old Infant. Vasc. Endovascular. Surg..

[49] Tetik O., Ozcem B., Calli A.O., Gurbuz A. (2010). True brachial artery aneurysm. Tex. Heart Inst. J..

[50] Bautista-Sánchez J., Cuipal-Alcalde J.D., Bellido-Yarlequé D., Rosadio-Portilla L., Gil-Cusirramos M. (2022). True Brachial Aneurysm in an Older Female Patient. A Case Report and Review of Literature. Ann. Vasc. Surg..

[51] Raja N., Kisku N., Gupta L., Geelani M.A. (2017). True Idiopathic Brachial Artery Aneurysm: A Rare Case of Surgical Emergency. Indian J. Vasc. Endovasc. Surg..

[52] Ghazi M.A., Khan A.M., Akram Y. (2006). Brachial Artery Aneurysm. Japan Med. Assoc. J..

[53] Parvin S.D., Bailey I.S. (1987). Brachial artery aneurysm in a five-year-old girl. Eur. J. Vasc. Surg.

[54] Greenberg J.I., Salamone L., Chang J., Harris E.J. (2012). Idiopathic true brachial artery aneurysm in an 18-month-old girl. J. Vasc. Surg..

[55] Palma F., Asciutto G., Usai M.V. (2024). Axillary artery aneurysms in pediatric patients: A narrative review. Vascular.

[56] Taketani S., Imagawa H., Kadoba K., Sawa Y., Sirakura R., Matsuda H. (1997). Idiopathic iliac arterial aneurysms in a child. J. Pediatr. Surg..

[57] Pagès O.N., Alicchio F., Keren B., Diallo S., Lefebvre F., Valla J.S., Poli-Merol M.L. (2008). Management of brachial artery aneurysms in infants. Pediatr. Surg. Int..

[58] Mowafy K.A., Soliman M.A. (2020). A Large Isolated True Brachial Artery Aneurysm in 6 months boy: Managed Surgically with Perfect Outcome. A Case Report. Int. J. Angiol. Vasc. Surg..

[59] Jones T.R., Frusha J.D., Stromeyer F.W. (1988). Brachial artery aneurysm in an infant: Case report and review of the literature. J. Vasc. Surg..

[60] Dawson J., Fitridge R. (2013). Update on aneurysm disease: Current insights and controversies: Peripheral aneurysms: When to intervene—Is rupture really a danger?. Prog. Cardiovasc. Dis..

[61] Schunn C.D., Sullivan T.M. (2002). Brachial arteriomegaly and true aneurysmal degeneration: Case report and literature review. Vasc. Med..

[62] He C., Wu X., Cao J., Fan X., Liu K., Liu B. (2013). Endovascular management of spontaneous axillary artery aneurysm: A case report and review of the literature. J. Med. Case Rep..

[63] Criado E., Marston W.A., Ligush J., Mauro M.A., Keagy B.A. (1997). Endovascular repair of peripheral aneurysms, pseudoaneurysms, and arteriovenous fistulas. Ann. Vasc. Surg..

[64] Papadoulas S., Stathopoulou M., Tsimpoukis A., Papageorgopoulou C., Nikolakopoulos K., Krinos N., Skandali A., Zampakis P., Mustaqe P., Dogjani A. (2025). Simultaneous Endovascular Abdominal Aortic Aneurysm Repair and Open Repair of Common Femoral Artery Aneurysm: Short Case Series and Current Review. J. Clin. Med..

[65] Tetik O., Yilik L., Besir Y., Can A., Ozbek C., Akcay A., Gurbuz A. (2005). Surgical treatment of axillary artery aneurysm. Tex. Heart Inst. J..

[66] Maskanakis A., Patelis N., Moris D., Tsilimigras D.I., Schizas D., Diakomi M., Bakoyiannis C., Georgopoulos S., Klonaris C., Liakakos T. (2018). Stenting of Subclavian Artery True and False Aneurysms: A Systematic Review. Ann. Vasc. Surg..

[67] Michaluk B.T., Deutsch E., Moufid R., Panetta T.F. (2009). Endovascular repair of an axillary artery pseudoaneurysm attributed to hyperextension injury. Ann. Vasc. Surg..

[68] Yılmaz F., Güvendi Şengör B., İzci S. (2021). Successful management of a brachial artery aneurysm with percutaneous intervention and one-month rivaroxaban therapy. Anatol. J. Cardiol..

[69] Yiğit G., Ozen S., Ozen A., İşcan H.Z. (2019). Isolated brachial artery aneurysm successfully treated with a covered stent in a patient with Behcet’s disease. Turk. Gogus. Kalp. Damar. Cerrahisi. Derg..

[70] Beregi J.P., Prat A., Willoteaux S., Vasseur M.A., Boularand V., Desmoucelle F. (1999). Covered stents in the treatment of peripheral arterial aneurysms: Procedural results and midterm follow-up. Cardiovasc. Interv. Radiol..

[71] Burnett H.F., Bledsoe J.H., Char F., Williams G.D. (1973). Abdominal aortic aneurysmectomy in a 17-year-old patient with Ehlers-Danlos syndrome: Case report and review of the literature. Surgery.

