# Supplementary material for: Idiopathic True Aneurysms of the Brachial Artery: A Short Case Series and Scoping Review
Source: J Clin Med. 2025 Dec 30;15(1):295. doi: 10.3390/jcm15010295 (PMC12786511; doi:10.3390/jcm15010295)
Supplement: Supplementary file 1 [file jcm-15-00295-s001.zip › jcm-4052903-supplementary.pdf]

Supplementary Material

Table S1. PRISMA Extension for Scoping Reviews (PRISMA-ScR): Checklist of items to include in reports of scoping reviews.

Section	Item	PRISMA-ScR Checklist Item	Reported in Manuscript
**TITLE**	1	Identify the report as a scoping review.	Title
**ABSTRACT**	2	Provide a structured summary that includes background, objectives, eligibility criteria, sources of evidence, charting methods, results, and conclusions.	Abstract
**INTRODUCTION**	3	Describe the rationale for the review in the context of what is already known.	Introduction
	4	Provide an explicit statement of the questions and objectives being addressed with reference to participants, concepts, and context.	Introduction
**METHODS**	5	Indicate whether a review protocol exists, and if so, where it can be accessed.	Not applicable (no protocol)
	6	Specify characteristics of the sources of evidence used as eligibility criteria.	Materials and Methods
	7	Describe all information sources in the search (e.g., databases) and the date of the most recent search.	Materials and Methods
	8	Present the full electronic search strategy for at least one database.	Materials and Methods
	9	State the process for selecting sources of evidence (screening and eligibility).	Materials and Methods
	10	Describe the methods for charting data from the included sources of evidence.	Materials and Methods
	11	Describe any critical appraisal of individual sources of evidence, if performed.	Not performed (scoping review)

**RESULTS**	12	Give numbers of sources of evidence screened, assessed for eligibility, and included in the review.	Results / Table 2
	13	Present characteristics of the sources of evidence included in the review.	Results / Table 2
	14	Present relevant data from the sources of evidence related to the review questions.	Results
**DISCUSSION**	15	Summarize the main results, including an overview of concepts, themes, and types of evidence available.	Discussion
	16	Discuss limitations of the scoping review process.	Limitations
	17	Provide a general interpretation of the results in relation to the review objectives and potential implications.	Discussion / Conclusion
**FUNDING**	18	Describe sources of funding for the included studies and for the scoping review itself.	Funding section
